# Locked Lateral Patellar Dislocation Reduced by Arthroscopic Procedure: A Case Report

**DOI:** 10.7759/cureus.76487

**Published:** 2024-12-27

**Authors:** Shunya Otani, Akira Tsujii, Kazunori Shimomura, Yasukazu Yonetani, Masayuki Hamada

**Affiliations:** 1 Department of Orthopaedic Surgery, Osaka University Graduate School of Medicine, Suita, JPN; 2 Department of Orthopaedic Surgery, Tokyo Women's Medical University, Tokyo, JPN; 3 Department of Sports Medical Biomechanics, Osaka University Graduate School of Medicine, Suita, JPN; 4 Department of Orthopaedic Surgery, Japan Community Health Care Organization Hoshigaoka Medical Center, Hirakata, JPN; 5 Department of Orthopaedic Surgery, Seifu Hospital, Sakai, JPN

**Keywords:** arthroscopic reduction, arthroscopic surgery, irreducible, lateral patellar dislocation, locked patellar dislocation, mpfl

## Abstract

Most cases of patellar dislocation can be reduced spontaneously or manually without sedation. To date, only one case of arthroscopic reduction for a lateral locked patellar dislocation has been reported, with a short follow-up period. Herein, we report the case of a 22-year-old man with a lateral locked patellar dislocation for whom we performed arthroscopic reduction and repair of the medial structure, which stabilized the patella medially. The patient was followed up for five years postoperatively and experienced no recurrence of patellar dislocation or difficulties in daily life.

## Introduction

Acute patellar dislocation is a relatively common injury which accounts for approximately 3% of all knee injuries [1]. Patellar dislocations often reduce spontaneously or can be reduced manually. Primary patellar dislocation is, therefore, commonly treated conservatively, whereas recurrent lateral patellar dislocation is preferentially treated by reconstructing the medial patellofemoral ligament (MPFL), which is known to be the primary restraint for lateral patellar dislocation [2].

Lateral locked patellar dislocation is a rare injury that is difficult to manually reduce [3], for which only a few cases have been reported. To date, most cases of closed or open reduction under general anesthesia have been reported. Only one case of arthroscopic reduction has been reported, with only short-term results being available [4]. Herein, we report a case of lateral locked patellar dislocation that was successfully treated with arthroscopic reduction and repair using medial patellar stabilizers, with long-term postoperative follow-up lasting several years.

## Case presentation

A 22-year-old man with an intellectual disability and a history of lateral patellar dislocation fell while farming and injured his left knee. He experienced severe pain and difficulty walking, prompting an urgent visit to another hospital. He was diagnosed with recurrent lateral patellar dislocation, but manual reduction attempts at the initial hospital were unsuccessful, and he was referred to our hospital the day after the injury.

**Figure 1:**
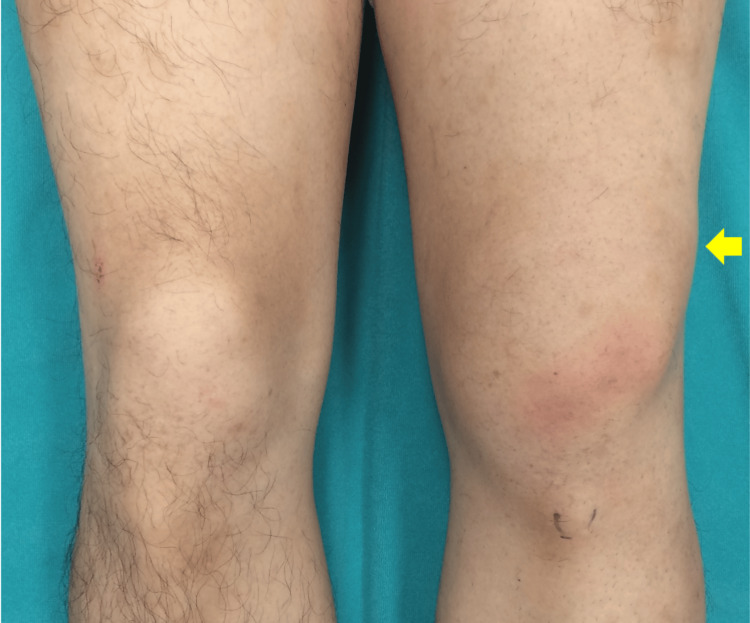
Preoperative appearance of the left knee showing lateral patellar dislocation with locking and swelling. Yellow arrow represented the dislocated patella.

Physical examination revealed that the knee was swollen with an obvious deformity, suggesting lateral displacement of the patella (Figure 1). Furthermore, the knee was fixed in an extended position and could not be flexed. Closed reduction was attempted again in our hospital, but this too was unsuccessful.

**Figure 2:**
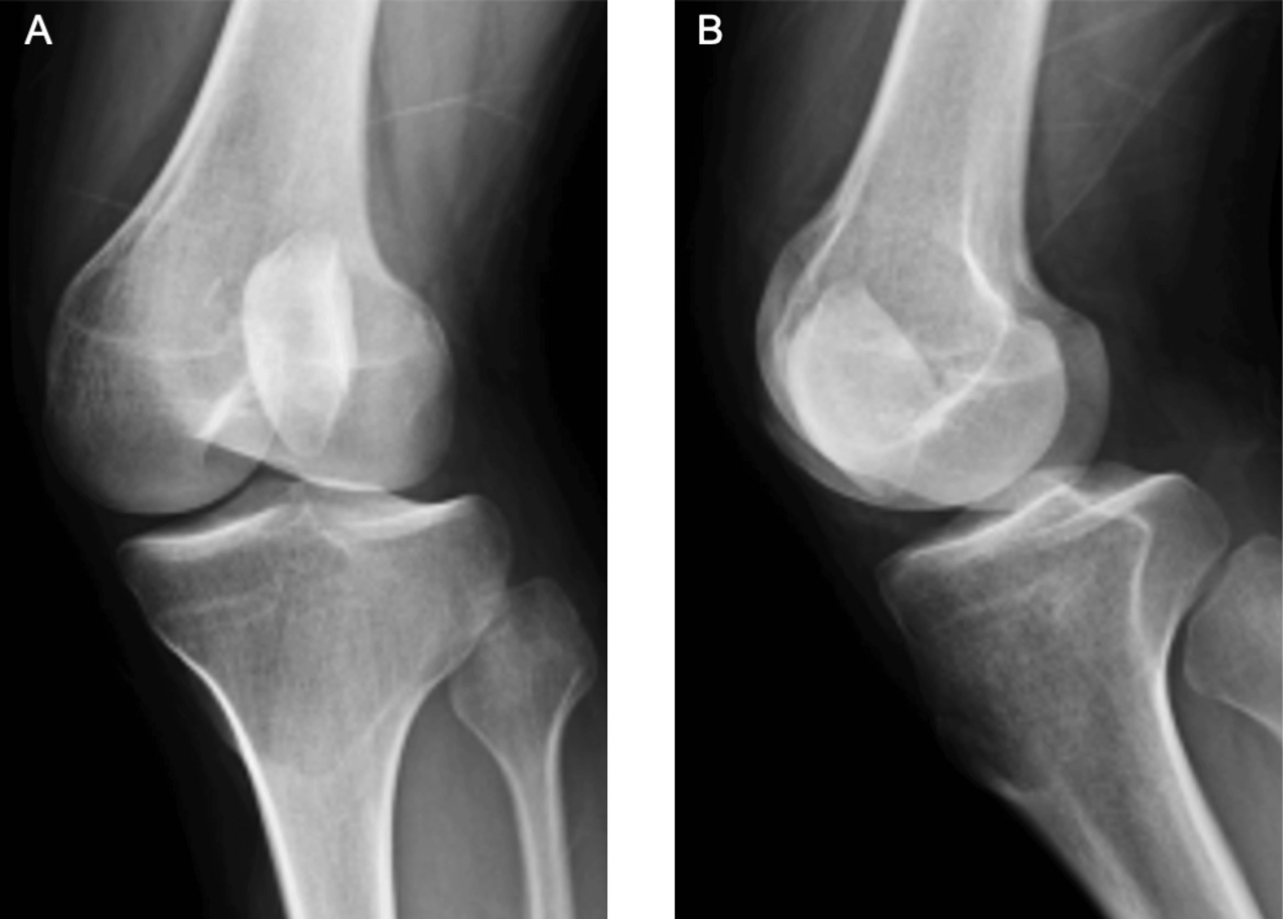
Preoperative plain radiographs: lateral patellar dislocation with patellar rotation. Skyline view could not be obtained due to difficulty in knee flexion.

**Figure 3:**
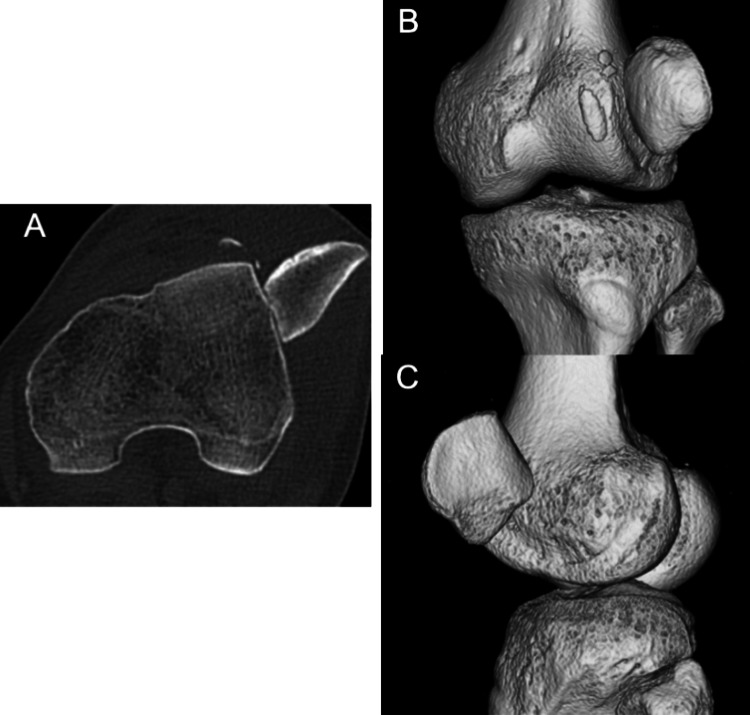
Preoperative multiplanar CT. (A) Axial view showed that the patella was axially rotated and locked outward on the lateral femoral condyle. (B-C) 3D CT images revealed that the patellar articular facet was facing outward. CT: computed tomography

Plain radiographs revealed lateral displacement of the patella (Figure 2A, 2B). A skyline view could not be obtained because of the difficulty in knee flexion. Computed tomography (CT) revealed small bone fragments, and the patella was axially rotated and locked outward on the lateral femoral condyle (Figure 3A). The tibial tubercle-trochlear groove (TT-TG) distance was 20.5 mm. 3D CT clearly showed that the patellar articular surface faced outwards (Figure 3B, 3C).

**Figure 4:**
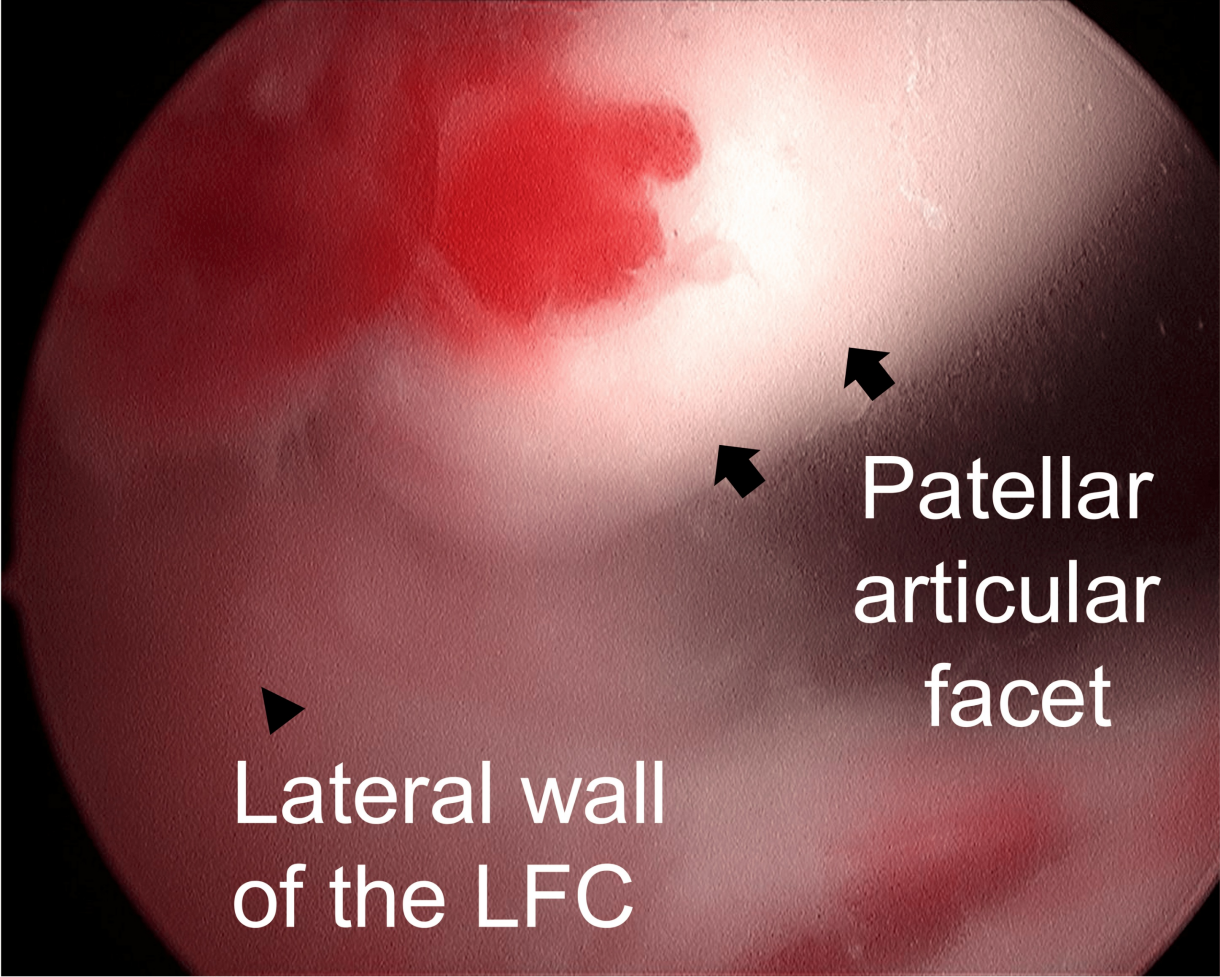
An arthroscopic image of the patella dislocated on the lateral wall of the LFC (arrowhead) and axially rotated. Arrows represented patellar articular facet. LFC: lateral femoral condyle

**Figure 5:**
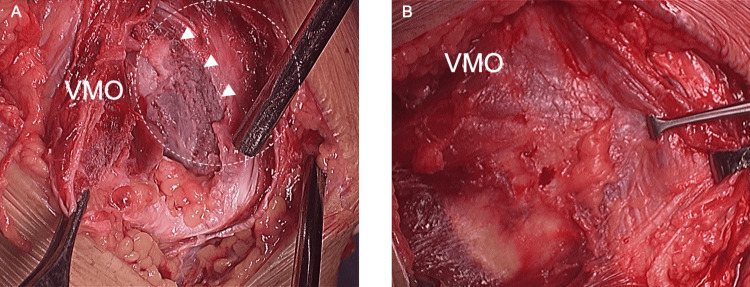
Gross appearance of the VMO muscle, MPFL, and MPTL. (A) All disrupted and detached from the periosteum contiguous to the patella (white dotted circle). White arrowheads represented the area to which the periosteum was originally attached. (B) The medial patellar stabilizers were repaired, and the medial retinaculum were reefed. VMO: vastus medialis oblique; MPFL: medial patellofemoral ligament; MPTL: medial patellotibial ligament

After confirming these findings with CT, another closed reduction attempt was made under general anesthesia, but it also failed. Therefore, knee arthroscopy was performed to reduce locked patellar dislocations. Arthroscopic evaluation revealed that the patella was dislocated laterally and extra-articularly and rotated axially (Figure 4). The medial edge of the patella was in contact with the lateral wall of the lateral femoral condyle, which hindered reduction. The elevatorium was inserted via the superolateral portal, and the patella was relocated by elevating it using like a shoehorn. Lateral release was performed inside the joint; however, patellar maltracking persisted. Subsequently, the ruptured medial structure was restored. An anteromedial skin incision was made and the vastus medialis oblique (VMO) muscle, MPFL, and medial patellotibial ligament (MPTL) attached to the patella were identified as disrupted and detached from the periosteum contiguous to the patella (Figure 5A). No. 2 FiberWire and TigerWire sutures (Arthrex, Naples, Florida, United States) were placed into both the VMO and MPFL using the Krackow suture technique, and the sutures were pulled out through 1.5-mm-diameter tunnels drilled transversely from the medial side to the lateral side of the patella and then fixed onto the lateral side of the patella at 45° of knee flexion. In addition, the remaining medial structures were advanced to the reef. Following the repair of the medial patellar stabilizers, patellar tracking improved. Finally, the medial capsule and retinaculum were tightly reefed (Figure 5B).

The knee was immobilized using an extension brace for two weeks. Subsequently, range of motion (ROM) exercises were introduced gradually. Weight-bearing restrictions were not applicable because of the patient's mental retardation; thus, full weight-bearing was permitted two weeks after surgery.

**Figure 6:**
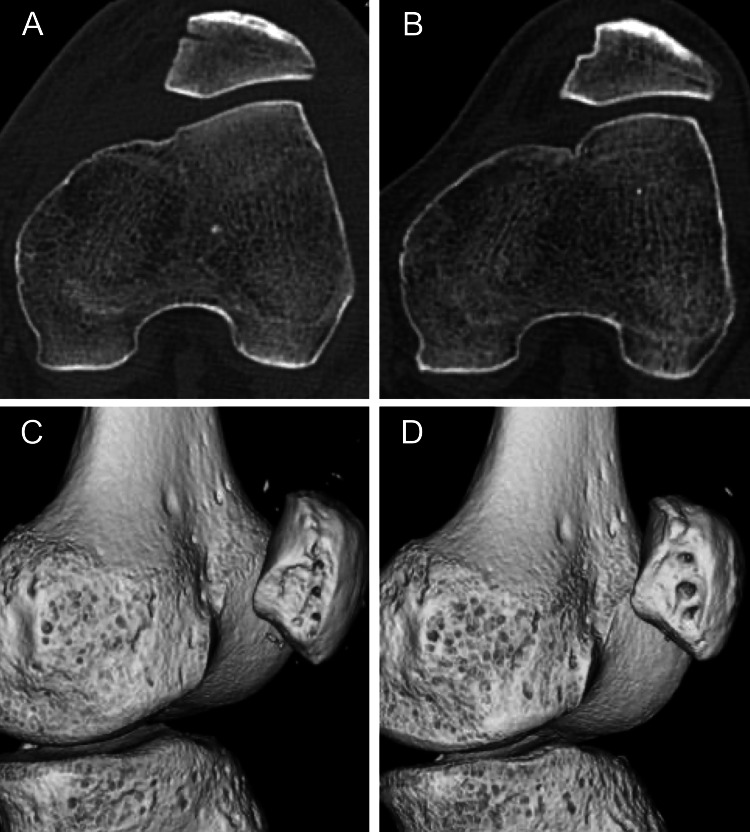
Follow-up CT images. (A, C) Postoperative one week. (B, D) Postoperative two years. CT: computed tomography

CT performed one week postoperatively revealed that the patella was repositioned and located in the femoral groove (Figure 6A), and its position was maintained for two years postoperatively (Figure 6B), while tunnel enlargement was observed (Figure 6C, 6D).

At the final follow-up (five years postoperatively), the patient had no limitation of ROM, no extension lag, and good clinical scores (Knee injury and Osteoarthritis Outcome Score (KOOS) score: pain, 100; symptoms, 93; activities of daily living (ADL), 97; sports, 85; quality of life (QOL), 87.5; International Knee Documentation Committee (IKDC) score, 85). There were no episodes of recurrent dislocation or osteoarthritic changes on the plain radiographs.

## Discussion

In general, lateral patellar dislocations can be easily reduced spontaneously or manually, although the locked lateral patellar dislocation described in this case is a rare injury. This rare dislocation pattern was first reported by Inman and Smart in 1941 [5], and only 24 cases have been reported to date. Only four cases (17%) were in patients over 50 years of age, and more than half of them (14/24, 58%) were in those aged <30 years, as in the present case. With regard to the mechanism of injury, 17 of 24 patients (71%) in the previous reports had obvious traumatic episodes, the most frequent being a direct blow to the knee. In addition, four cases of injuries caused by falls have been reported, as in the current case. In contrast, seven cases with no obvious traumatic episodes have been reported. Three of these patients (43%) reported a history of previous dislocation similar to our case [6-8].

Three reduction techniques have been reported for the management of this condition: closed, open, and arthroscopic techniques. Closed reduction is generally the first attempted procedure and is often performed under general anesthesia for locked patellar dislocation [9-12]. However, repeated closed reduction for locked patellar dislocation should be avoided because of the risk of additional damage to the cartilage and soft tissue [4]. Open reduction has been the most frequently performed procedure for lateral locked patellar dislocation, performed in 16 of 24 cases (67%) in previous reports. Arthroscopic reduction has only been reported by Teixeira et al. [4]. The advantage of this approach is that it is minimally invasive and can be used to evaluate other articular lesions. Moreover, patellar tracking can be assessed directly, enabling the addition of a lateral release, which can further be performed arthroscopically. In the current case, arthroscopy revealed that the patellar tracking after lateral release remained laterally shifted and improved after repair of the medial patellar stabilizers.

Regarding the cause of patellar irreducibility, a previous report postulated that this is due to a strong direct external force from the medial side of the knee that causes the patella to significantly overcome the lateral femoral condyle, which in turn causes the patella to rotate in the axial direction [5]. Other reports have described avulsion fractures of the medial side of the patella or osteophyte formation in the femur, which may make reduction difficult [9,13]. In the present case, CT images revealed axial rotation of the patella with evidence of avulsed bone fragments and consequent impaction of the patella on the lateral wall of the lateral femoral condyle, which indicates difficulty in reduction [13]. Moreover, in this case, the chronic dysfunction of the MPFL, which has been reported to be the primary soft tissue restraint to lateral patellar dislocation [14,15], was considered to exist due to a previous recurrent dislocation. Detachment of the VMO, which also plays an important role in medial patellar stability [16,17], results in the loss of medial traction. In the present study, all medial structures ruptured, which might have made patellar reduction difficult.

In previous reports, medial structural repair after patellar reduction has been performed in 13 of 17 cases (76%) that required open or arthroscopic reduction. In the remaining three cases, no additional procedures were performed following reduction without recurrent dislocation after surgery, and in one case, total knee arthroplasty was performed. However, as a meta-analysis showed that MPFL repair significantly reduced the recurrence rate compared with nonoperative treatment [18], medial structure repairs should be performed. In this case, no recurrent dislocation was observed after five years of follow-up, and the clinical outcome was deemed successful, suggesting that repair of the primary medial structures, including the MPFL and VMO, could be useful for such severe dislocations.

A summary of the previous reports is shown in Table 1.

**Table 1:** Review of previously reported cases of locked patellar dislocation. TKA: total knee arthroplasty; F/U: follow-up

Author	Year	Age	Sex	Mechanism	History	Reduction method	Fracture	Additional treatment	F/U
Inman and Smart [5]	1941	43	M	Direct blow	NA	Open	Yes	Medial repair	8 months
Moed and Morawa [19]	1981	18	M	Atraumatic (while walking)	No	Closed	No	-	3 weeks
Benjamin and Percy [6]	1984	38	F	Atraumatic (climb onto the roof)	Yes (2)	Open	No	Medial capsular reefing and lateral release	6 months
Corso et al. [20]	1992	16	M	Direct blow	No	Open	No	Medial repair and plication	NA
Hackl et al. [21]	1999	53	F	Twisting and direct blow	No	Open	Yes	Medial repair (anchor) and lateral release	1 year
Gorczyca and Grau [22]	2000	13	M	Direct blow	NA	Open	No	Medial suture repair	6 months
ElMaraghy et al. [23]	2002	30	F	Hyperflexion	No	Open	Yes	Medial retinacular repair	10 months
Phaltankar and Bridle [24]	2002	66	F	Atraumatic	No	Open	Yes	TKA	NA
Sherman and Yu [25]	2004	28	M	Fall to ground	No	Closed	No	-	NA
Abdelhalim et al. [10]	2007	8	M	Fall from staircase	No	Closed	No	-	NA
Feibel et al. [13]	2007	66	F	Fall on ice	No	Open	Yes	No	6 weeks
Huang [21]	2008	12	M	Direct blow	No	Closed	No	-	NA
Michels et al. [26]	2008	16	F	Atraumatic (while dancing)	No	Open	Yes	Lateral release	1 year.
Yang et al. [12]	2011	19	M	Direct blow	NA	Closed	No	-	26 months
Louw and Jansen van Rensburg [27]	2012	17	F	Twisting during karate	NA	Open	Yes	Medial plication and coverage lateral defect	18 months
Lowe et al. [28]	2012	50	M	Fall from hill	No	Open	Yes	Medial repair	6 weeks
Yerimah et al. [29]	2013	21	M	Direct blow	NA	Open	Yes	Medial repair	NA
Grewal et al. [9]	2014	32	F	Direct blow	No	Closed	Yes	-	NA
Devgan et al. [8]	2016	14	M	Atraumatic (while running)	Yes (1)	Open	No	Medial plication and lateral release	18 months
Higgins and Khalfaoui [7]	2016	32	M	Atraumatic (move to next seat)	Yes (2)	Open	No	Medial retinacular repair	3 months
Delagrammaticas and Cordes [30]	2016	32	F	Twisting	No	Open	Yes	No	1 year
Teixeira et al. [4]	2018	23	M	Direct blow	NA	Arthroscopic	No	No	6 months
Chaudhary et al. [31]	2021	24	F	Atraumatic (while walking)	NA	Open	No	Medial soft tissue repair	1 year
Aflatooni and McKay [3]	2023	17	M	Twisting during football	Yes (3)	Closed	No	-	NA

## Conclusions

To the best of our knowledge, this is the first report of midterm follow-up after arthroscopic reduction and medial patellar stabilizer repair for lateral locked patellar dislocation. In general, open or arthroscopic reduction is recommended for this type of dislocation because of the risk of additional iatrogenic injury associated with closed reduction by force. Arthroscopic reduction is useful because it is less invasive and allows visualization, including patellar tracking, from inside the joint.
